# Prospective Validation and Usability Evaluation of a Mobile Diagnostic App for Obstructive Sleep Apnea

**DOI:** 10.3390/diagnostics14222519

**Published:** 2024-11-11

**Authors:** Pedro Amorim, Daniela Ferreira-Santos, Marta Drummond, Pedro Pereira Rodrigues

**Affiliations:** 1 Sleep and Non-Invasive Ventilation Unit, São João University Hospital, 4200-319 Porto, Portugal; 2 Center for Health Technology and Services Research (CINTESIS), 4200-450 Porto, Portugal; 3 Faculty of Medicine, University of Porto (FMUP), 4200-319 Porto, Portugal; 4 School of Health—P.Porto, 4200-072 Porto, Portugal; 5 INESC TEC—Institute for Systems and Computer Engineering, Technology and Science, 4200-465 Porto, Portugal

**Keywords:** obstructive sleep apnea, diagnosis, mobile applications, Bayesian network, artificial intelligence, polysomnography

## Abstract

**Background/Objectives**: Obstructive sleep apnea (OSA) classification relies on polysomnography (PSG) results. Current guidelines recommend the development of clinical prediction algorithms in screening prior to PSG. A recent intuitive and user-friendly tool (OSABayes), based on a Bayesian network model using six clinical variables, has been proposed to quantify the probability of OSA. Our aims are (1) to validate OSABayes prospectively, (2) to build a smartphone app based on the proposed model, and (3) to evaluate app usability. **Methods**: We prospectively included adult patients suspected of OSA, without suspicion of other sleep disorders, who underwent level I or III diagnostic PSG. Apnea–hypopnea index (AHI) and OSABayes probabilities were obtained and compared using the area under the ROC curve (AUC [95%CI]) for OSA diagnosis (AHI ≥ 5/h) and higher severity levels (AHI ≥ 15/h) prediction. We built the OSABayes app on ‘App Inventor 2’, and the usability was assessed with a cognitive walkthrough method and a general evaluation. **Results**: 216 subjects were included in the validation cohort, performing PSG levels I (34%) and III (66%). OSABayes presented an AUC of 83.6% [77.3–90.0%] for OSA diagnosis and 76.3% [69.9–82.7%] for moderate/severe OSA prediction, showing good response for both types of PSG. The OSABayes smartphone application allows one to calculate the probability of having OSA and consult information about OSA and the tool. In the usability evaluation, 96% of the proposed tasks were carried out. **Conclusions**: These results show the good discrimination power of OSABayes and validate its applicability in identifying patients with a high pre-test probability of OSA. The tool is available as an online form and as a smartphone app, allowing a quick and accessible calculation of OSA probability.

## 1. Introduction

Obstructive sleep apnea (OSA) is a common sleep-related breathing disorder characterized by partial or complete upper airway collapse, repeated throughout sleep [1,2]. This may result in fragmented sleep, intermittent hypoxia, intrathoracic pressure swings, and increased sympathetic nerve activity [2,3]. Thus, patients may report witnessed apneas or snoring, but also nocturia, excessive sleepiness, non-restful sleep, morning headaches, decreased concentration, memory loss, or irritability [1,3,4,5].

OSA affects about 926 million adults worldwide [6], and untreated patients may be at increased risk of developing cardiovascular disease, metabolic dysregulation, diabetes, or neuropsychiatric conditions [1,7,8]. OSA symptoms and their association with other diseases reinforce the importance of early and accurate diagnosis [7]. However, available data suggest that many cases of OSA remain undiagnosed and untreated [6].

The diagnosis of OSA requires symptoms coupled with a predominantly obstructive apnea–hypopnea index (AHI) ≥ 5 events per hour (/h) in polysomnography (PSG) [9]. Since a full PSG (PSG I) cannot be performed for all patients due to the high prevalence of OSA, costs, waiting lists, and the great demand for tests, portable recording devices (PSG III) have been developed to allow laboratory-equivalent diagnosis, although with fewer channels [3]. These devices are particularly interesting in patients with a high probability of moderate to severe OSA pre-test [10,11].

Further, current American Academy of Sleep Medicine (AASM) guidelines recommend the development of clinical prediction algorithms in screening before PSG [1]. In this context, some studies have tried to show that, beyond questionnaires, machine learning methods can also be used in OSA [11,12,13,14]. In addition, clinical decision support applications also have considerable potential to improve access to care and its quality [15].

One of these methods is Bayesian networks, a model of a joint probability distribution over a set of variables that allows a general and versatile approach to capturing and reasoning with uncertainty [16,17]. Based on this, Ferreira-Santos et al. [18] created an auxiliary diagnostic method—OSABayes—that could support the decision to perform PSG based on risk and diagnostic factors. The authors studied 38 variables in a sample of individuals with suspected OSA, pre-PSG I, selecting six variables with a univariate significant association with the outcome. To build the model, a tree-augmented naïve Bayes classifier was used. This approach allows a more natural description of the relationships between variables, admitting up to two dependencies for each factor, which can be important since, for example, not only OSA causes nocturia but age as well [4]. So, an intuitive and user-friendly form was created with these six variables (gender, age, neck circumference (NC), craniofacial abnormalities (CFA), witnessed apneas, and nocturia) that can be used in the primary care setting to quantify the probability of having OSA and help to refer only the suspected patients to sleep laboratories [19].

OSABayes was validated following an internal cross-validation approach, and an online form is already available. Thus, our aims are (1) to validate OSABayes prospectively, (2) to build a smartphone app based on the proposed model, and (3) to evaluate app usability.

## 2. Materials and Methods

This study followed STROBE recommendations for observational studies [20].

### 2.1. Patients

We have prospectively included adult patients suspected of having OSA referred to perform a PSG I or III at the sleep laboratory of São João University Hospital between December 2019 and March 2020. Pregnant women, patients with nocturnal oxygen supplementation, or performing therapeutic studies with positive airway pressure therapy, positional therapy, or oral appliances were excluded.

### 2.2. Data Collection and Preprocessing

Prior to PSG, all patients were observed by a sleep physiologist who collected OSABayes variables and other variables to characterize the cohort, namely demographic variables (body mass index (BMI)), clinical history (Epworth sleepiness scale (ESS), snoring, morning headaches, non-restful sleep), and comorbidities (hypertension, diabetes, dyslipidemia, cardiac pathology, stroke, and depression).

The OSABayes probability was obtained by filling out the form. The PSG review and analysis were performed by an experienced sleep physiologist that was blind to those probabilities.

### 2.3. Polysomnography

We carried out validation in both PSG I and PSG III to find out how the tool behaves in each of the diagnostic tests.

PSG III was performed at home with portable equipment (Embletta^®^ MPR [Embla Systems], Kanata, ON, Canada) that records respiratory flow through a nasal cannula, respiratory effort with inductance plethysmography belts, pulse oximetry, and body position. An effective diagnosis was considered whenever the AASM criteria were met [9] with a high-quality recording time of more than 4 h [1].

PSG I was performed in the sleep laboratory of São João University Hospital, with the recording of electroencephalogram, electrooculogram, chin and legs electromyography, electrocardiogram, respiratory flow through the nasal cannula and thermistor, respiratory effort with inductance plethysmography belts, pulse oximetry, and body position. PSG I was performed using Embla^®^ S4500 [Embla Systems], Kanata, ON, Canada or Alice^®^ 6 [Respironics] equipment, Pittsburgh, PA, USA.

Sleep epochs were defined according to AASM Scoring Manual version 2.5 criteria [21], and since all subjects in the study had suspected OSA—signs/symptoms or associated diseases—OSA diagnosis was confirmed with AHI values ≥ 5/h in PSG I or III.

### 2.4. Smartphone App

The OSABayes app (Version 1.0) was based on the form already available online, providing an alternative platform for use. The application was built on the ‘App Inventor 2’ (https://appinventor.mit.edu/, accessed on 1 May 2022)—an open-source application provided by Massachusetts Institute of Technology (MIT) that allows the creation of software applications for Android [22].

The OSABayes smartphone application calculates the probability of having OSA and consults information about OSA (such as prevalence, risk factors, diagnosis, or treatment) and the tool (such as data on model derivation and validation).

### 2.5. Smartphone App Evaluation

The usability evaluation of the app was also carried out by the expected end users. This evaluation aimed to assess the ability and ease of carrying out some intended actions and evaluate the visibility, consistency, efficiency of use, and design, among other features. The usability was assessed using a cognitive walkthrough method (CWM) and a general evaluation.

For the CWM—a structured method that allows the evaluation of the application’s usability with learning through exploration—a to-do list was built with some tasks that the user should try to perform with little formal instruction. Ultimately, they must classify whether they could perform the task with ease or difficulty or could not perform it [23].

General usability was assessed through a questionnaire supported by the Portuguese version of the System Usability Scale (SUS) [24]. The SUS consists of 10 statements punctuated between ‘1—totally disagree’ and ‘5—totally agree’, with the score adjusted from 0 to 100 [25].

After the two evaluations were carried out, a few more general questions were asked to collect potential end-users’ opinions about the OSABayes application and to hear their possible ideas for improvement.

### 2.6. Statistical Analysis

Categorical variables were described with absolute and relative frequencies and compared using the Chi-squared or Fisher’s exact test. Continuous variables were characterized by means and standard deviations (unless otherwise specified). Comparisons of continuous variables were performed using the independent *T*-test or Mann–Whitney. Statistical significance was defined at 5%, and the statistical analysis was performed using R software (version 4.1.2).

The area under the receiver operating characteristics curve (AUC) assessed the model’s accuracy. AHI and OSABayes probabilities were obtained and compared using the AUC [95%CI] for OSA diagnosis (AHI ≥ 5/h) and for higher severity levels (AHI ≥ 15/h) of OSA prediction. The model’s accuracy was also assessed for the diagnosis of OSA performed by PSG I and PSG III. A cutoff value was chosen to assess the model’s discriminative capacity. This value was obtained after assessing the ROC curve for OSA diagnosis (AHI ≥ 5/h), aiming at a sensitivity ≥ 95%.

### 2.7. Ethics

The Ethics Committee of São João University Hospital (CES 335-19) approved the study following the Declaration of Helsinki.

## 3. Results

### 3.1. Validation Cohort

The validation cohort had 216 patients with OSA suspicion. The mean (sd) age was 57 (13), with 55% of males. A total of 73 subjects performed PSG I (34%) and 143 subjects performed PSG III (66%). OSA diagnosis was present in 177 (82%), from which 78 (44%) were categorized as mild, 56 (32%) moderate, and 43 (24%) severe. Table 1 describes the validation cohort, comparing normal ones to OSA patients.

Among OSABayes variables, only the CFA did not differ between OSA patients and normal subjects. Male gender, age, NC, witnessed apneas, and nocturia were significantly higher in patients with OSA. In addition, snoring, hypertension, diabetes, and dyslipidemia were also significantly higher in OSA patients compared to normal subjects. The percentage of subjects with increased BMI (44 vs. 56% in OSA group, *p* = 0.151) and ESS (57 vs. 40% in OSA group, *p* = 0.064) did not differ significantly between groups. Non-restful sleep was superior in the group without OSA (74% vs. 50% in the OSA group, *p* ≤ 0.05).

The subjects who performed PSG I or PSG III also exhibit some differences. Male gender, NC, and snoring (83% vs. 97% in PSG III, *p* ≤ 0.001) were significantly higher in PSG III. In contrast, the percentage of patients with depression was significantly higher in PSG I. The percentage of patients with OSA was considerably higher in the PSG III group (66% vs. 90% in PSG III, *p* < 0.001).

### 3.2. Model Validation

**Figure 1:**
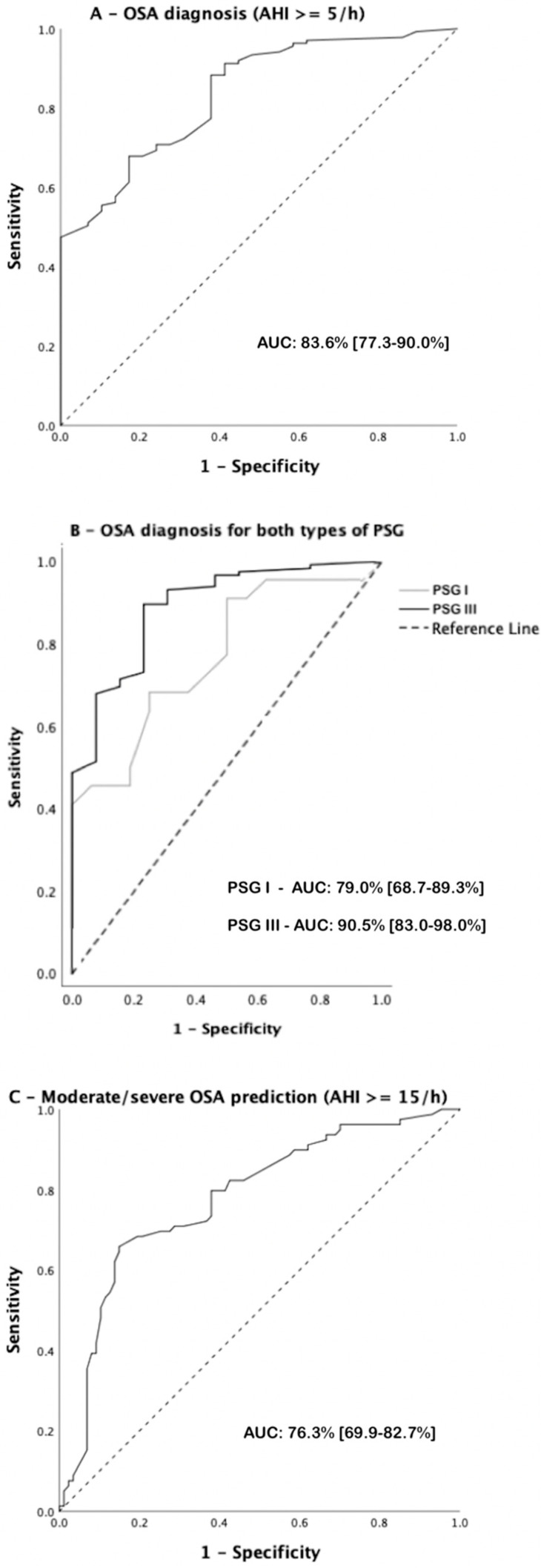
Receiver operating characteristics analyses and area under the curve values for OSA diagnosis (**A**), for diagnosis in both types of PSG (**B**), and for moderate/severe OSA prediction (**C**). AUC values are shown with their 95% confidence intervals.

The OSABayes was validated following an external approach. ROC analysis and the respective AUCs, along with their 95% confidence intervals, are shown in Figure 1. For AHI ≥ 5/h, the AUC was 83.6% [77.3–90.0%], being slightly higher in PSG III (90.5% [83.0–98.0%]) than in PSG I (79.0% [68.7–89.3%]), although without statistical significance (*p* = 0.117). For AHI ≥ 15/h, the AUC was 76.3% [69.9–82.7%].

### 3.3. Cutoff Definition

After an evaluation of the coordinates of the ROC curve for OSA diagnosis, a cutoff value of 4.0% was defined, with a sensitivity > 95%. This cutoff can be used in a primary care setting to quantify the probability of having OSA and help to refer patients to sleep laboratories. Table 2 shows the discriminatory capacity of the model in different settings.

Generally, a cutoff of 4.0% had a sensitivity of 96.6% and a specificity of 41.0%. The diagnostic odds ratio was 19.74 and the diagnostic accuracy was 86.6%, with an F-score of 0.91. For AHI ≥ 5/h in each PSG, specificity was slightly lower in PSG I. In PSG III, positive post-test odds of 19.26 was found, with a diagnostic accuracy of 92.3% and a diagnostic odds ratio of 31.26. For moderate/severe OSA prediction, the cutoff of 4.0% had a general sensitivity value of 97.0%. Although having low specificity (27.4%), it had a negative predictive value of 91.5% and negative post-test odds of 0.09.

As expected, AHI values were higher in patients with OSABayes > 4.0% (20.4/h vs. 7.1/h, *p* > 0.001).

### 3.4. OSABayes Evaluation with Metrics Beyond AHI

Beyond AHI, oxygen variables also have differences. In the group with OSABayes higher than the cutoff, the mean SpO2 value (92.9% vs. 95.2%, *p* = 0.001) and the minimum oxygen saturation (81.9% vs. 89.2%) are lower. On the other hand, this same group presents higher values of saturation time below 90% (10.9% vs. 1.0%, *p* < 0.001) and desaturation index (21.5/h vs. 7.4/h).

If we apply the Baveno classification [26]—which focuses more on symptoms and comorbidities and not on the AHI—we see that of patients with OSABayes probability greater than the cutoff, 74% have associated symptoms and/or comorbidities. Furthermore, most of the suspected patients not indicated to perform PSG by the OSABayes belonged to groups A (68%) and B (28%) of the Baveno classification. Only one patient was classified as Baveno C, and none as Baveno D.

### 3.5. Smartphone App

The OSABayes app consists of six main screens and nine frames, that is, platforms that are integrated into the same screen but that appear and disappear according to the user’s options and may look like different screens.

The six screens are: Intro, Menu, Calculate, Learn more, OSA, and OSABayes. Moreover, the nine frames are: Results (integrated into the ‘Calculate’ screen), Definition, Prevalence, Risk factors, Diagnosis and Treatment (integrated into the ‘OSA’ screen), Info, Studies, and Team (integrated into the ‘OSABayes’ screen).

**Figure 2:**
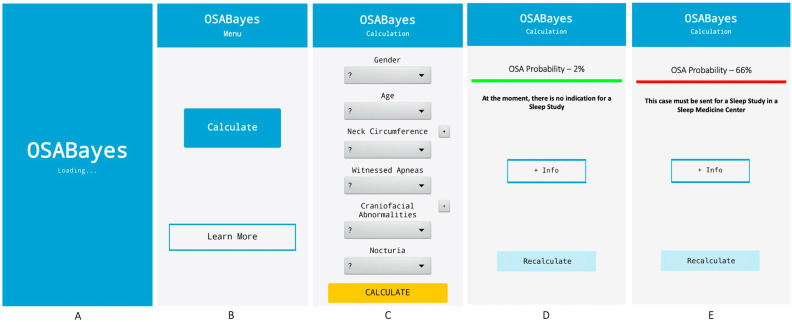
OSABayes application screens—(**A**) (initial screen), (**B**) (main menu), (**C**) (calculation screen), (**D**) (OSABayes result screen—negative example), (**E**) (OSABayes result screen—positive example).

The app’s navigation always starts with the ‘Intro’ screen, which gives automatic access to the ‘Menu’ screen. The practice ends on one of the three final screens: ‘Calculate’, ‘OSA’, and ‘OSABayes’. Figure 2 shows some of the OSABayes app screens.

### 3.6. Smartphone App Evaluation

Eight physicians with no previous contact with the application conducted a usability assessment. Five (62.5%) were male, with a median (interquartile range) age of 48 (36–57). 6 (75%) used the Android operating system daily, and 2 (25%) used iOS.

The assessment results through the CWM were globally positive: 82% of the tasks were performed successfully and efficiently, 14% were performed with difficulty, and only 4% of the tasks failed to be performed. The most general usability evaluation showed an average of 90.4—higher than the 68, defined as a good result, and above 84, which is needed to classify as a maximum level (A+) [25,27].

## 4. Discussion

OSABayes is an intuitive and user-friendly tool based on a Bayesian network, which allows clinicians to quantify the probability of having OSA with six clinical and demographic variables.

The tool is available as an online form and as a smartphone app, allowing a quick and accessible calculation of OSABayes probability. Furthermore, the usability assessment demonstrated the ease of executing the intended actions and the consistency and efficiency in using the application.

Our validation data shows the excellence of OSABayes in diagnosing OSA (AHI ≥ 5/h), with a global AUC of 83.6% [77.3–90.0%]. The model also appears to have a high discriminatory power regardless of the type of PSG used, with slightly better results in PSG III than in PSG I.

The fact that OSABayes responds well in patients who performed PSG III means that it can be useful in a larger number of patients because, as we saw: (1) the model was created to improve the initial screening of OSA in primary health care and to help ensure a more efficient hospital patients’ referral, and (2) PSG III is an available test for the initial screening of the patients with a moderate to high probability of the disease. In addition, the AUC for PSG I has an acceptable value of 77.0% [62.1–91.9%].

As the relevance of this study lies in the validation of a model that allows only the most likely OSA cases to be referred for screening, it was important to define a cutoff above which a patient should be considered for PSG. Setting a cutoff value of 4.0% demonstrated a sensitivity > 95% for the diagnosis of OSA (in general and in each PSG), with an overall diagnostic accuracy of 86.6% and an F-score of 0.91, ensuring test quality.

Even if we look at metrics other than the AHI, such as oximetry or the Baveno classification, we can see that this screening tool allows us to distinguish the patients with higher end-organ impact from the others and reinforces how OSABayes can be useful in distinguishing who benefits from PSG.

According to our validation, we expect about 11% false positives for both PSGs, which, despite being undesirable, are an improvement compared to all patients at risk referred for sleep consultation and PSG. Nonetheless, we could rule out 41% of healthy patients, which would reduce the burden of unnecessary consultations and waiting lists for PSG.

Compared to other screening tools widely tested for OSA, OSABayes has a slightly higher AUC value (83.6% vs. 79.6% and 82.6% in STOP-Bang [28,29] and NoSAS [30], respectively), although very similar. In addition to these promising results, OSABayes has the advantage of being shorter than the STOP-Bang and the Berlin questionnaire [31,32]. Furthermore, it is advantageous that the OSABayes calculation is automatic and can be performed both online and in the smartphone app, even with missing variables.

This study has a few limitations. First, few patients have performed PSG I (*n* = 73). Second, the prevalence of OSA in our cohort is high; thus, the results may not apply to the general population, where the prevalence of OSA is lower. Also, we acknowledge that the model’s specificity is not as high as we would like. Even so, the specificity has values > 35% for the diagnosis of OSA in general and in each PSG. In the future, it is essential to understand the behavior of OSABayes in other settings and to study the economic impact that this tool can have.

## 5. Conclusions

OSABayes can be a valuable tool in daily clinical practice for identifying patients with a high probability of the disease. The model is based on clinical and demographic variables, which are easy and quick to acquire and work even without one or more variables [33]. The form is intuitive and user-friendly and can be applied quickly during a medical appointment to quantify the probability of having OSA, helping to refer patients to sleep laboratories. Our results showed the excellent performance of OSABayes in both types of PSG, reducing the number of unnecessary exams, which can help to reduce waiting times and health costs.

## Figures and Tables

**Table 1:** Descriptive analysis of the patients included in the validation cohort, comparing OSA with normal subjects. The results shown are the *n* (%) and *p* value results of the Chi-squared test, unless specified otherwise.

	Total (*n* = 216)	Normal Subjects (*n* = 39)	OSA (*n* = 177)	*p* Value
Male gender	119 (55%)	12 (31%)	107 (61%)	**<0.001**
Age				
Mean (SD)	57 (13)	48 (11)	59 (12)	**<0.001** ^#^
<40	21 (10%)	9 (23%)	12 (7%)	**0.001**
40–54	66 (30%)	20 (51%)	46 (26%)	
55–69	92 (43%)	9 (23%)	83 (47%)	
>70	37 (17%)	1 (3%)	36 (20%)	
NC				
Median (IQR)	40 (37–43)	36 (33–39)	41 (38–44)	**<0.001** ^+^
Increased	88 (41%)	5 (13%)	83 (48%)	**<0.001**
Witnessed apneas	120 (56%)	12 (31%)	108 (62%)	**<0.001**
Nocturia	123 (58%)	14 (36%)	109 (62%)	**0.003**
CFA	25 (12%)	2 (5%)	23 (13%)	0.264 *
BMI				
Mean (SD)	31 (6)	28 (5)	31 (6)	**<0.001** ^#^
Obese	116 (54%)	17 (44%)	99 (56%)	0.151
ESS				
Median (IQR)	9 (5–15)	11 (4–14)	9 (5–15)	0.729 ^+^
Increased	77 (43%)	20 (57%)	57 (40%)	0.064
Snoring	166 (92%)	26 (72%)	140 (97%)	**<0.001**
Morning headache	69 (39%)	20 (56%)	49 (35%)	**0.024**
Non-restful sleep	94 (55%)	26 (74%)	68 (50%)	**0.010**
Hypertension	95 (55%)	11 (31%)	84 (61%)	**0.002**
Diabetes	39 (23%)	1 (3%)	38 (28%)	**<0.001** *
Dyslipidemia	79 (46%)	10 (29%)	69 (50%)	**0.023**
Cardiac pathology	21 (12%)	2 (3%)	19 (14%)	0.054 *
Stroke	11 (6%)	2 (6%)	9 (7%)	0.606 *
Depression	48 (28%)	10 (29%)	38 (28%)	0.922

OSA: obstructive sleep apnea; SD: standard deviation; IQR: interquartile range; NC: neck circumference; CFA: craniofacial abnormalities; BMI: body mass index; ESS: Epworth sleepiness scale. ^#^ Independent T test, ^+^ Mann–Whitney test; * Fisher’s exact test. Bold text indicates a *p* ≤ 0.05.

**Table 2:** Diagnostic accuracy measures of the model with a cutoff of 4.0% in different settings: diagnosis of OSA with PSG I or PSG III, as well as in the prediction of moderate/severe cases. Values shown in parentheses are 95% CIs.

	AHI ≥ 5/h			AHI ≥ 15/h		
	Total	PSG III	PSG I	Total	PSG III	PSG I
Sensitivity (%)	96.6 [92.8–98.4]	96.9 [92.3–99.2]	95.8 [85.9–99.5]	97.0 [91.4–99.4]	96.3 [89.6–99.2]	94.4 [72.7–99.9]
Specificity (%)	41.0 [25.6–57.9]	50.0 [23.0–77.0]	36.0 [17.8–57.5]	27.4 [19.5–36.4]	24.2 [14.2–36.7]	30.9 [19.1–44.8]
PPV (%)	88.1 [82.8–91.9]	94.7 [91.4–96.8]	74.2 [68.0–79.5]	53.1 [50.1–55.9]	62.4 [58.9–65.8]	30.9 [26.6–35.6]
NPV (%)	72.7 [52.8–86.5]	63.6 [36.9–84.0]	81.3 [51.0–94.7]	91.5 [77.1–97.1]	83.4 [60.2–94.3]	94.4 [70.9–99.2]
Dx accuracy (%)	86.6 [81.3–90.8]	92.3 [86.7–96.1]	75.3 [63.8–84.7]	59.3 [52.4–65.9]	65.0 [56.6–72.8]	46.6 [34.8–58.6]
Dx OR	19.74 [18.57–20.92]	31.26 [29.81–32.70]	12.52 [10.90–14.14]	12.20 [10.98–13.43]	8.31 [7.02–9.60]	7.54 [5.45–9.63]
LR+	1.64 [1.26–2.13]	2.09 [1.15–3.28]	1.50 [1.11–2.02]	1.34 [1.19–1.50]	1.27 [1.10–1.47]	1.37 [1.11–1.69]
LR−	0.08 [0.03–0.20]	0.06 [0.02–0.19]	0.12 [0.03–0.50]	0.11 [0.03–0.35]	0.15 [0.05–0.51]	0.18 [0.03–1.26]
Odds Post+	7.43 [6.51–8.35]	19.26 [16.18–22.33]	2.87 [2.34–3.40]	1.13 [1.08–1.18]	1.66 [1.49–1.83]	0.45 [0.33–0.56]
Odds Post−	0.38 [0.31–0.44]	0.57 [0.49–0.65]	0.23 [0.13–0.33]	0.09 [0.05–0.13]	0.20 [0.13–0.27]	0.06 [0.01–0.11]
F-score	0.91 [0.88–0.94]	0.98 [0.96–1.00]	0.77 [0.70–0.84]	0.55 [0.52–0.58]	0.65 [0.61–0.69]	0.33 [0.23–0.43]

AHI: apnea–hypopnea index; PPV: positive predictive value; NPV: negative predictive value; Dx accuracy: diagnostic accuracy; Dx OR: diagnostic odds ratio; LR+: positive likelihood ratio; LR−: negative likelihood ratio; Odds Post+: positive post-test odds; Odds Post−: negative post-test odds.

## Data Availability

Data supporting the reported results are unavailable due to privacy and ethical restrictions.
